# {Bis[4-(2-pyrid­yl)pyrimidin-2-yl]sulfane}dichloridocobalt(II)

**DOI:** 10.1107/S1600536809022636

**Published:** 2009-06-17

**Authors:** Hai-Bin Zhu, Lei Li, Jun-Feng Ji

**Affiliations:** a School of Chemistry and Chemical Engineering, Southeast University, Nanjing 211189, People’s Republic of China

## Abstract

The asymmetric unit of the title compound, [CoCl_2_(C_18_H_12_N_6_S)], contains one half-mol­ecule situated on a twofold rotation axis which passes through the Co and S atoms. The metal centre is in a distorted octahedral CoCl_2_N_4_ coordination with the Cl atoms in the axial positions. In the crystal structure, inter­molecular C—H⋯Cl inter­actions help to establish the packing.

## Related literature

For coordination compounds with bis­(4-pyridin­yl)sulfane, see: Jung *et al.* (1998 ▶); Ni & Vittal (2001 ▶).
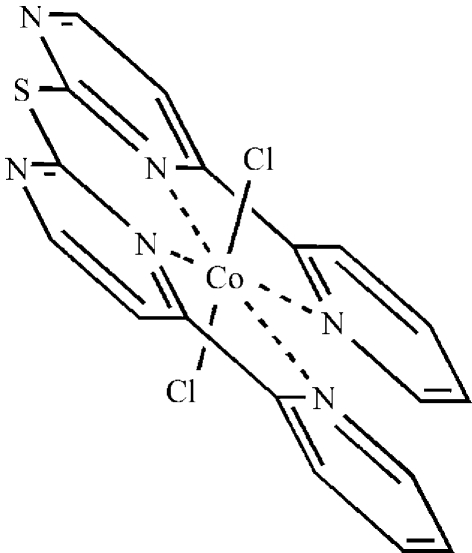


## Experimental

### 

#### Crystal data

[CoCl_2_(C_18_H_12_N_6_S)]
*M* *_r_* = 474.24
Monoclinic, 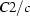

*a* = 14.685 (3) Å
*b* = 10.325 (2) Å
*c* = 13.376 (3) Å
β = 112.339 (3)°
*V* = 1875.9 (7) Å^3^
*Z* = 4
Mo *K*α radiation
μ = 1.33 mm^−1^
*T* = 298 K
0.20 × 0.18 × 0.12 mm

#### Data collection

Bruker APEXII CCD area-detector diffractometer
Absorption correction: multi-scan (*SADABS*; Bruker, 2001 ▶) *T*_min_ = 0.884, *T*_max_ = 0.920 (expected range = 0.819–0.853)
6015 measured reflections
2302 independent reflections
1526 reflections with *I* > 2σ(*I*)
*R*_int_ = 0.141

#### Refinement

*R*[*F* ^2^ > 2σ(*F* ^2^)] = 0.046
*wR*(*F* ^2^) = 0.077
*S* = 1.00
2302 reflections
128 parameters
H-atom parameters constrained
Δρ_max_ = 0.51 e Å^−3^
Δρ_min_ = −0.68 e Å^−3^

### 

Data collection: *APEX2* (Bruker, 2007 ▶); cell refinement: *SAINT-Plus* (Bruker, 2007 ▶); data reduction: *SAINT-Plus*; program(s) used to solve structure: *SHELXS97* (Sheldrick, 2008 ▶); program(s) used to refine structure: *SHELXL97* (Sheldrick, 2008 ▶); molecular graphics: *SHELXTL* (Sheldrick, 2008 ▶); software used to prepare material for publication: *SHELXTL*.

## Supplementary Material

Crystal structure: contains datablocks I, global. DOI: 10.1107/S1600536809022636/at2809sup1.cif

Structure factors: contains datablocks I. DOI: 10.1107/S1600536809022636/at2809Isup2.hkl

Additional supplementary materials:  crystallographic information; 3D view; checkCIF report

## Figures and Tables

**Table 1:** Hydrogen-bond geometry (Å, °)

*D*—H⋯*A*	*D*—H	H⋯*A*	*D*⋯*A*	*D*—H⋯*A*
C2—H2*A*⋯Cl1^i^	0.93	2.63	3.546 (3)	170
C3—H3*A*⋯Cl1^ii^	0.93	2.73	3.584 (3)	154
C7—H7*A*⋯Cl1^iii^	0.93	2.76	3.580 (4)	148

Symmetry codes: (i) 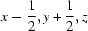; (ii) 
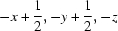
; (iii) 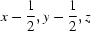.

**Fig. 1.**
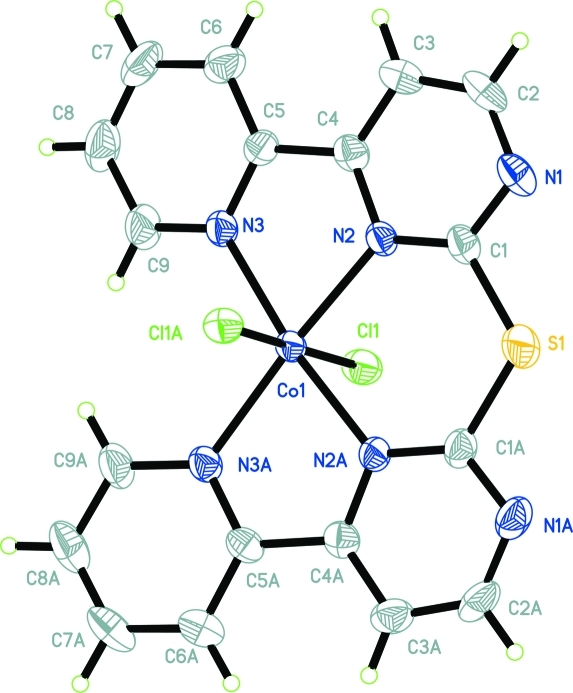
The molecular structure of the title compound showing the atomic numbering and 40% probability displacement ellipsoids [symmetry code: (A) -x, y, 1/2 - z.]

## supplementary crystallographic information

### Comment

Organic ligand of bis(4-pyridinyl)sulfane has been employed to construct some intriguing metal–orgainc frameworks (MOFs) (Jung *et al.*,1998; Ni & Vittal, 2001). Herein, we report the molecular structure of a mononuclear Co^II^ coordination complex (I) with bis(4-(pyridin-2-yl)pyrimidin-2-yl)sulfane.

In compound (I), the cobalt(II) ion is six-coordinated by four N atoms in equatorial position and two Cl atoms in apical position (Fig. 1). The Co—N bond lengths are 2.102 (2) Å and 2.130 (2) Å and the Co—Cl bond distance is 2.4363 (9) Å. In the crystal, intermolecular C—H···Cl interactions help to establish the packing.

### Experimental

To a solution of CoCl_2 _(0.1 mmol) in MeOH (10 ml) was added a solution of bis(4-(pyridin-2-yl)pyrimidin-2-yl)sulfane (0.1 mmol) in CH_2_Cl_2_ (5 ml). The mixture was stirred for 30 min, then filtered. The mother liquid was stood at ambient temperature for two days to give the orange crystals.

### Refinement

All H atoms were positioned geometrically and allowed to ride on their parent atoms, with C—H = 0.93 Å and *U*_iso_(H) = 1.2*U*_eq_(C) for aromatic H atoms.

### Crystal data

**Table:**

[CoCl_2_(C_18_H_12_N_6_S)]	*F*(000) = 956
*M**_r_* = 474.24	*D*_x_ = 1.679 Mg m^−^^3^
Monoclinic, *C*2/*c*	Mo *K*α radiation, λ = 0.71073 Å
Hall symbol: -C 2yc	Cell parameters from 2302 reflections
*a* = 14.685 (3) Å	θ = 2.3–25.5°
*b* = 10.325 (2) Å	µ = 1.33 mm^−^^1^
*c* = 13.376 (3) Å	*T* = 298 K
β = 112.339 (3)°	Block, orange
*V* = 1875.9 (7) Å^3^	0.20 × 0.18 × 0.12 mm
*Z* = 4	

### Data collection

**Table:**

Bruker APEXII CCD area-detector diffractometer	2302 independent reflections
Radiation source: fine-focus sealed tube	1526 reflections with *I* > 2σ(*I*)
graphite	*R*_int_ = 0.141
φ and ω scans	θ_max_ = 28.4°, θ_min_ = 2.5°
Absorption correction: multi-scan (*SADABS*; Bruker, 2001)	*h* = −17→19
*T*_min_ = 0.884, *T*_max_ = 0.920	*k* = −6→13
6015 measured reflections	*l* = −17→17

### Refinement

**Table:**

Refinement on *F*^2^	Primary atom site location: structure-invariant direct methods
Least-squares matrix: full	Secondary atom site location: difference Fourier map
*R*[*F*^2^ > 2σ(*F*^2^)] = 0.046	Hydrogen site location: inferred from neighbouring sites
*wR*(*F*^2^) = 0.077	H-atom parameters constrained
*S* = 1.00	*w* = 1/[σ^2^(*F*_o_^2^) + (0.01*P*)^2^] where *P* = (*F*_o_^2^ + 2*F*_c_^2^)/3
2302 reflections	(Δ/σ)_max_ = 0.001
128 parameters	Δρ_max_ = 0.51 e Å^−^^3^
0 restraints	Δρ_min_ = −0.67 e Å^−^^3^

### Special details

**Table:**

Geometry. All e.s.d.'s (except the e.s.d. in the dihedral angle between two l.s. planes) are estimated using the full covariance matrix. The cell e.s.d.'s are taken into account individually in the estimation of e.s.d.'s in distances, angles and torsion angles; correlations between e.s.d.'s in cell parameters are only used when they are defined by crystal symmetry. An approximate (isotropic) treatment of cell e.s.d.'s is used for estimating e.s.d.'s involving l.s. planes.
Refinement. Refinement of *F*^2^ against ALL reflections. The weighted *R*-factor *wR* and goodness of fit *S* are based on *F*^2^, conventional *R*-factors *R* are based on *F*, with *F* set to zero for negative *F*^2^. The threshold expression of *F*^2^ > σ(*F*^2^) is used only for calculating *R*-factors(gt) *etc*. and is not relevant to the choice of reflections for refinement. *R*-factors based on *F*^2^ are statistically about twice as large as those based on *F*, and *R*- factors based on ALL data will be even larger.

### Fractional atomic coordinates and isotropic or equivalent isotropic displacement parameters (Å^2^)

**Table:**

	*x*	*y*	*z*	*U*_iso_*/*U*_eq_	
Co1	0.5000	0.21623 (4)	0.2500	0.03357 (15)	
Cl1	0.52209 (5)	0.21891 (6)	0.07848 (5)	0.04298 (19)	
S1	0.5000	0.57242 (8)	0.2500	0.0646 (4)	
N3	0.37405 (17)	0.09651 (17)	0.17599 (17)	0.0358 (5)	
N2	0.38470 (16)	0.35147 (16)	0.19288 (16)	0.0327 (5)	
C4	0.2933 (2)	0.3022 (2)	0.15111 (19)	0.0353 (6)	
C1	0.3909 (2)	0.4812 (2)	0.2002 (2)	0.0382 (7)	
N1	0.3163 (2)	0.56366 (19)	0.16953 (19)	0.0473 (6)	
C5	0.2875 (2)	0.1579 (2)	0.14019 (19)	0.0350 (6)	
C3	0.2114 (2)	0.3803 (3)	0.1175 (2)	0.0456 (7)	
H3A	0.1482	0.3460	0.0884	0.055*	
C2	0.2275 (2)	0.5119 (3)	0.1289 (2)	0.0500 (8)	
H2A	0.1733	0.5669	0.1070	0.060*	
C6	0.1984 (2)	0.0954 (2)	0.0938 (2)	0.0472 (7)	
H6A	0.1398	0.1419	0.0695	0.057*	
C8	0.2854 (3)	−0.1011 (3)	0.1176 (2)	0.0586 (9)	
H8A	0.2869	−0.1906	0.1108	0.070*	
C9	0.3722 (2)	−0.0329 (2)	0.1618 (2)	0.0523 (9)	
H9A	0.4315	−0.0777	0.1825	0.063*	
C7	0.1980 (3)	−0.0383 (3)	0.0841 (2)	0.0566 (9)	
H7A	0.1390	−0.0837	0.0552	0.068*	

### Atomic displacement parameters (Å^2^)

**Table:**

	*U*^11^	*U*^22^	*U*^33^	*U*^12^	*U*^13^	*U*^23^
Co1	0.0269 (3)	0.0223 (2)	0.0436 (3)	0.000	0.0045 (2)	0.000
Cl1	0.0342 (4)	0.0443 (4)	0.0449 (4)	0.0018 (3)	0.0089 (3)	0.0000 (3)
S1	0.0532 (8)	0.0242 (5)	0.0984 (10)	0.000	0.0086 (7)	0.000
N3	0.0360 (14)	0.0272 (10)	0.0381 (12)	−0.0012 (10)	0.0072 (10)	0.0015 (9)
N2	0.0333 (13)	0.0253 (10)	0.0349 (12)	0.0029 (10)	0.0077 (10)	0.0012 (8)
C4	0.0382 (17)	0.0384 (14)	0.0244 (13)	0.0011 (13)	0.0064 (12)	0.0006 (10)
C1	0.0434 (18)	0.0290 (12)	0.0390 (15)	0.0063 (12)	0.0121 (14)	0.0017 (11)
N1	0.0512 (18)	0.0374 (12)	0.0472 (15)	0.0170 (12)	0.0117 (13)	0.0037 (10)
C5	0.0354 (16)	0.0382 (13)	0.0280 (13)	−0.0061 (13)	0.0083 (13)	0.0016 (11)
C3	0.0343 (17)	0.0556 (17)	0.0403 (16)	0.0079 (15)	0.0067 (14)	0.0014 (13)
C2	0.047 (2)	0.0529 (17)	0.0445 (18)	0.0276 (16)	0.0118 (16)	0.0072 (13)
C6	0.0395 (18)	0.0564 (17)	0.0403 (16)	−0.0103 (15)	0.0092 (14)	0.0023 (13)
C8	0.076 (3)	0.0356 (14)	0.0502 (19)	−0.0182 (17)	0.0082 (19)	−0.0005 (13)
C9	0.058 (2)	0.0286 (13)	0.0580 (19)	−0.0056 (14)	0.0079 (17)	−0.0030 (12)
C7	0.059 (2)	0.0570 (18)	0.0466 (18)	−0.0322 (17)	0.0116 (17)	−0.0023 (14)

### Geometric parameters (Å, °)

**Table:**

Co1—N2^i^	2.102 (2)	C1—N1	1.323 (3)
Co1—N2	2.1017 (19)	N1—C2	1.320 (4)
Co1—N3	2.130 (2)	C5—C6	1.377 (4)
Co1—N3^i^	2.130 (2)	C3—C2	1.378 (3)
Co1—Cl1^i^	2.4363 (9)	C3—H3A	0.9300
Co1—Cl1	2.4363 (9)	C2—H2A	0.9300
S1—C1	1.757 (3)	C6—C7	1.386 (3)
S1—C1^i^	1.757 (3)	C6—H6A	0.9300
N3—C5	1.335 (3)	C8—C7	1.354 (5)
N3—C9	1.349 (3)	C8—C9	1.377 (4)
N2—C4	1.343 (3)	C8—H8A	0.9300
N2—C1	1.343 (3)	C9—H9A	0.9300
C4—C3	1.374 (4)	C7—H7A	0.9300
C4—C5	1.497 (3)		
			
N2^i^—Co1—N2	96.73 (11)	N1—C1—N2	126.5 (3)
N2^i^—Co1—N3	172.65 (7)	N1—C1—S1	107.47 (18)
N2—Co1—N3	77.25 (8)	N2—C1—S1	126.1 (2)
N2^i^—Co1—N3^i^	77.25 (8)	C2—N1—C1	116.0 (2)
N2—Co1—N3^i^	172.65 (7)	N3—C5—C6	123.5 (2)
N3—Co1—N3^i^	109.05 (11)	N3—C5—C4	115.3 (2)
N2^i^—Co1—Cl1^i^	91.57 (6)	C6—C5—C4	121.3 (3)
N2—Co1—Cl1^i^	87.57 (6)	C4—C3—C2	116.8 (3)
N3—Co1—Cl1^i^	92.37 (6)	C4—C3—H3A	121.6
N3^i^—Co1—Cl1^i^	88.39 (6)	C2—C3—H3A	121.6
N2^i^—Co1—Cl1	87.57 (6)	N1—C2—C3	123.1 (3)
N2—Co1—Cl1	91.57 (6)	N1—C2—H2A	118.5
N3—Co1—Cl1	88.39 (6)	C3—C2—H2A	118.5
N3^i^—Co1—Cl1	92.37 (6)	C5—C6—C7	118.6 (3)
Cl1^i^—Co1—Cl1	178.70 (3)	C5—C6—H6A	120.7
C1—S1—C1^i^	115.15 (17)	C7—C6—H6A	120.7
C5—N3—C9	117.0 (2)	C7—C8—C9	120.3 (3)
C5—N3—Co1	115.55 (15)	C7—C8—H8A	119.9
C9—N3—Co1	127.4 (2)	C9—C8—H8A	119.9
C4—N2—C1	115.9 (2)	N3—C9—C8	122.1 (3)
C4—N2—Co1	116.07 (15)	N3—C9—H9A	118.9
C1—N2—Co1	127.82 (19)	C8—C9—H9A	118.9
N2—C4—C3	121.7 (2)	C8—C7—C6	118.4 (3)
N2—C4—C5	115.4 (2)	C8—C7—H7A	120.8
C3—C4—C5	122.9 (3)	C6—C7—H7A	120.8
			
N2—Co1—N3—C5	−5.56 (17)	C1^i^—S1—C1—N1	177.9 (2)
N3^i^—Co1—N3—C5	170.5 (2)	C1^i^—S1—C1—N2	−3.15 (18)
Cl1^i^—Co1—N3—C5	81.40 (18)	N2—C1—N1—C2	0.7 (4)
Cl1—Co1—N3—C5	−97.53 (18)	S1—C1—N1—C2	179.6 (2)
N2—Co1—N3—C9	174.6 (2)	C9—N3—C5—C6	2.2 (4)
N3^i^—Co1—N3—C9	−9.30 (19)	Co1—N3—C5—C6	−177.6 (2)
Cl1^i^—Co1—N3—C9	−98.4 (2)	C9—N3—C5—C4	−176.2 (2)
Cl1—Co1—N3—C9	82.7 (2)	Co1—N3—C5—C4	3.9 (3)
N2^i^—Co1—N2—C4	−177.8 (2)	N2—C4—C5—N3	1.7 (3)
N3—Co1—N2—C4	6.51 (17)	C3—C4—C5—N3	179.4 (2)
Cl1^i^—Co1—N2—C4	−86.47 (17)	N2—C4—C5—C6	−176.9 (2)
Cl1—Co1—N2—C4	94.50 (17)	C3—C4—C5—C6	0.9 (4)
N2^i^—Co1—N2—C1	−3.04 (17)	N2—C4—C3—C2	−0.2 (4)
N3—Co1—N2—C1	−178.8 (2)	C5—C4—C3—C2	−177.8 (2)
Cl1^i^—Co1—N2—C1	88.2 (2)	C1—N1—C2—C3	−0.5 (4)
Cl1—Co1—N2—C1	−90.8 (2)	C4—C3—C2—N1	0.2 (4)
C1—N2—C4—C3	0.3 (4)	N3—C5—C6—C7	0.4 (4)
Co1—N2—C4—C3	175.7 (2)	C4—C5—C6—C7	178.8 (2)
C1—N2—C4—C5	178.1 (2)	C5—N3—C9—C8	−3.3 (4)
Co1—N2—C4—C5	−6.5 (3)	Co1—N3—C9—C8	176.5 (2)
C4—N2—C1—N1	−0.6 (4)	C7—C8—C9—N3	1.7 (5)
Co1—N2—C1—N1	−175.4 (2)	C9—C8—C7—C6	1.0 (5)
C4—N2—C1—S1	−179.38 (19)	C5—C6—C7—C8	−2.0 (4)
Co1—N2—C1—S1	5.9 (3)		

Symmetry codes: (i) −*x*+1, *y*, −*z*+1/2.

### Hydrogen-bond geometry (Å, °)

**Table:**

*D*—H···*A*	*D*—H	H···*A*	*D*···*A*	*D*—H···*A*
C2—H2A···Cl1^ii^	0.93	2.63	3.546 (3)	170
C3—H3A···Cl1^iii^	0.93	2.73	3.584 (3)	154
C7—H7A···Cl1^iv^	0.93	2.76	3.580 (4)	148

Symmetry codes: (ii) *x*−1/2, *y*+1/2, *z*; (iii) −*x*+1/2, −*y*+1/2, −*z*; (iv) *x*−1/2, *y*−1/2, *z*.
